# The Impact of Landing Complexity and Knee Taping on Stability: A Continuous Kinetics and Kinematics Analysis †

**DOI:** 10.3390/sports13120431

**Published:** 2025-12-03

**Authors:** Kendra Taryn Szeles, Andrew Green

**Affiliations:** Department of Sport and Movement Studies, Faculty of Health Science, University of Johannesburg, Johannesburg 2028, South Africa

**Keywords:** injury prevention, Statistical Parametric Mapping, strapping, Biomechanics

## Abstract

Landing biomechanics are strongly influenced by task complexity, yet the combine effects of different landing demands and taping on stability, kinetics, and kinematics remain unclear. Nineteen female athletes performed 40 cm drop landings (DL), drop jump landings (DJL), and countermovement jumps (CMJ) under four knee taping conditions: no tape (NT), rigid tape (RT), dynamic tape (DT), and kinesio tape (KT). Stability indices were compared across tasks and taping conditions. Continuous landing-phase biomechanics were analysed using SPM1d repeated measures ANOVA (*p* < 0.05). SPM1d revealed significant GRF differences between landing tasks (0–3%, *p* = 0.026; 15–25%, *p* < 0.001), with DT (*p* = 0.02) and KT (*p* = 0.03) reducing peak landing forces in the DJL compared to DL. The DL showed greater biomechanical stability overall, with better dynamic postural stability index (DPSI) across all taping conditions. However, TTS was significantly shorter in the DJL than the DL in RT (*p* = 0.005), DT and KT (*p* = 0.037). Significant joint kinematic differences were found between tasks and taping, particularly at the ankle, knee, and hip. Landing complexity influences joint loading and stability. Knee taping may attenuate impact forces and improve stabilisation during complex tasks, suggesting a potential role in enhancing movement efficiency and supporting injury-prevention.

## 1. Introduction

Landing from a jump requires rapid deceleration of the body’s centre of mass (CoM) to restore stability [1]. Anticipatory muscle activation before ground contact provides feedforward neuromuscular control, while the lower limbs act as a spring-damper system, absorbing impact forces through joint flexion and elastic tissue loading [1,2]. Inefficient landing strategies are strongly associated with lower limb injuries, particularly when elevated ground reaction forces (GRF), knee valgus collapse, or delayed stabilisation occur [1,2,3].

Jump landing tasks such as the drop landing (DL), drop jump landing (DJL), and countermovement jump (CMJ) are widely used to assess lower limb function, neuromuscular control, and injury risk, with each task differing in complexity and muscular demand [4]. Task complexity in landing is commonly described in terms of the number of coordinated movement phases, the degree of neuromuscular recruitment, and the attentional demands required for successful completion [5,6,7,8]. Specifically, the DL is considered the least complex landing task, as it requires only a single phase of deceleration and stabilisation, which consequently produce greater hip, knee, and ankle flexion and higher GRF due to rapid CoM deceleration [7,9,10,11]. By contrast, the DJL and CMJ are classified as more complex dual-task movements, involving a preceding propulsion phase and subsequent landing [1,8,12]. This added complexity alters landing mechanics by requiring greater muscle preactivation and elastic energy reuse to attenuate impact, whereas the DL relies predominantly on joint motion [7]. Task demands are further influenced by attentional focus, where external focus instructions promote greater knee dominant energy dissipation, whereas dual-task landings increase attentional load and reduce postural stability [5,6,13]. As a result, DJL and CMJ have been shown to delay balance recovery and increase variability in GRF, alongside greater knee abduction and ankle adduction, compared with the simpler DL [14,15,16].

Knee taping is a common external intervention used to support landing control and mitigate injury risk. Rigid tape (RT) provides mechanical restriction to unwanted joint displacement, while dynamic tape (DT) contributes elastic resistance and recoil that may aid deceleration and load absorption [17,18]. Kinesio tape (KT) acts primarily through neurophysiological mechanisms, enhancing proprioceptive input and joint position sense to support postural control [19,20]. Previous studies have reported mixed findings on the effects of taping on GRF, knee valgus, and joint kinematics during landing [21,22]. These discrepancies may stem from differences in task design (single versus dual task landings), variations in taping technique, and the use of discrete rather than continuous outcome measures. This variability underlines the importance of examining multiple task conditions within a unified experimental framework across tasks of varying complexity.

Traditional biomechanical analyses have typically focused on discrete time points, such as peak or minimum values, which may overlook adaptations occurring across the full movement cycle [23]. One-dimensional Statistical Parametric Mapping (SPM1d) addresses this limitation by analysing kinematic and kinetic data across the entire time-normalised cycle [19,24]. This approach enables the detection of subtle, phase-specific changes that discrete methods may miss. SPM1d has been increasingly applied to landing tasks, such as DL, DJL, and CMJ, to examine continuous joint angles [25,26] and GRFs [27]. For example, it has revealed fatigue-related reductions in propulsive force during repeated CMJs [28], decreased ankle plantarflexion and inversion in response to ankle RT during jumping [29], and reduced knee abduction at ground contact in response to knee KT during DJL [19]. This makes SPM1d particularly valuable when evaluating interventions such as knee taping, where changes in stability or joint motion may not occur at a single instant but across extended phases of the landing cycle.

Despite these insights, few studies have directly compared postural stability across DL, DJL, and CMJ using continuous analysis techniques, and none to date have investigated the effects of joint taping across these tasks. Therefore, this study aimed to compare the landing phases of the DL, DJL, and CMJ and investigate the effects of different taping conditions on lower limb stability, kinetics, and kinematics using SPM1d. It was hypothesised that the DJL and CMJ would demonstrate increased neuromuscular demand and altered joint kinematics compared to the DL, and that taping would modulate joint motion and potentially enhance postural control across all tasks.

## 2. Materials and Methods

### 2.1. Participants

A sample size estimation was performed in G*Power (Version 3.1.9.7). Assuming a large effect (f = 0.4; η^2^ ≈ 0.5), with a significance level of α = 0.05 and statistical power set at 0.95, the calculation indicated that at least 15 participants would be required.

A total of nineteen healthy female netball players (mean age: 23.4 ± 2.9 years; height: 169.0 ± 8.0 cm; body mass: 62.9 ± 10.3 kg) volunteered for the study. Written informed consent was obtained from all participants in advance, and ethical clearance was granted by the Faculty of Health Sciences Ethics Committee (REC-1760-2022) before data collection began.

### 2.2. Procedure

**Figure 1:**
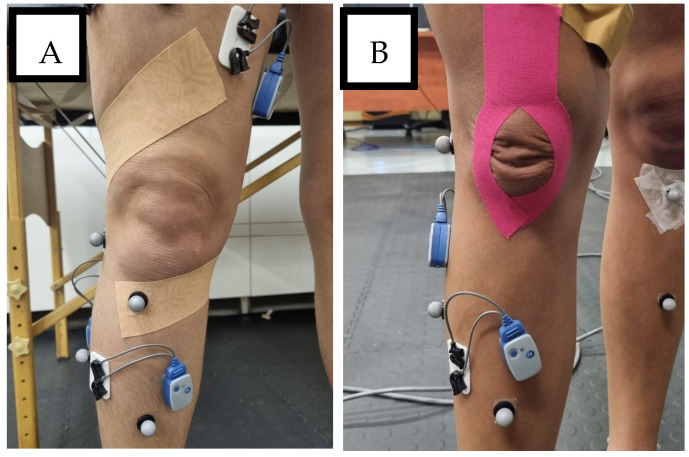
Taping techniques—(**A**) Mulligan’s Taping Technique, (**B**) “Y” Formation Technique.

Each participant completed three jumping tasks—drop landing (DL), drop jump landing (DJL), and countermovement jump (CMJ)—under four taping conditions applied in randomised order: no tape (NT), rigid tape (RT), dynamic tape (DT), and kinesio tape (KT) following the procedures used in the study by Szeles and Green [30] (Figure 1). Rigid and dynamic tapes were applied using Mulligan’s technique [31,32], while KT was applied using the Y-formation method [33]. All applications were performed by the same practitioner to maintain consistency.

To minimise fatigue and learning effects, the order of taping conditions and landing tasks was randomised across participants. Each participant was provided with three practice trials per task before data collection to ensure familiarisation. Rest periods of one minute between jumps and five minutes between taping conditions were enforced to minimise fatigue accumulation.

For the DL, participants stepped from a 40 cm box with hands on hips and landed toe-first with each foot on separate force plates, maintaining balance for three seconds [34]. The DJL followed the same initial procedure but required an immediate vertical countermovement jump upon landing before a second landing on the force plates [34]. For the CMJ, participants performed self-selected countermovement jumps with hands on hips, aiming to maximise take-off height by executing a rapid eccentric-concentric sequence [35]. Any attempt not conforming to protocol was discarded and repeated until three valid trials were collected in each condition.

### 2.3. Biomechanical Analysis

Lower-limb kinematics were captured using the CGM 2.5 model with a 10-camera Vicon Vero 2.2 system (Vicon Motion Systems Ltd., Oxford, UK) operating at 200 Hz. Ground reaction forces were simultaneously collected at 1000 Hz via two synchronised multi-axis force platforms (Bertec Corporation, Columbus, OH, USA). Three-dimensional marker trajectories and force data were processed in Nexus software (https://nexussoftwares.com/, accessed on 30 July 2025) (Vicon Motion Systems Ltd., Oxford, UK), which was also used for calibration, tracking, and data export. Kinematic signals were low-pass filtered with a fourth-order, zero-lag Butterworth filter at a 6 Hz cut-off frequency.

### 2.4. Data Analysis

The landing profile (0–100% of ground contact) was divided into three phases: loading (1–6%), attenuation (7–26%), and control (27–100%) [36]. Ground contact was defined as the instant when vertical ground reaction force (GRF) exceeded 0.01 body weight. For each condition, three valid landings were time-normalised, averaged, and analysed. Only the terminal landing from each task (the DL, second contact of the DJL, and the CMJ landing) was included, up to the point where participants regained postural stability.

Time to stabilisation (TTS) was calculated using the method outlined by Ross and Guskiewicz [37], where the point of intersection between normalised vertical GRF and a third-order polynomial fit during the control phase was identified. Dynamic postural stability was assessed via the Dynamic Postural Stability Index (DPSI), derived from GRF fluctuations in the anterior–posterior (AP), medial-lateral (ML), and vertical (V) directions over three seconds following initial contact [38].

Joint kinematic data were exported from Nexus (Vicon Motion Systems Ltd., Oxford, UK) and processed in MATLAB version 2023b (Mathworks, Natick, MA, USA) with custom algorithms for Statistical Parametric Mapping (SPM1d) analysis.

### 2.5. Statistical Analysis

Following normality testing, non-parametric data (TTS) were analysed using a two-way ANOVA and Friedman’s test. Parametric variables, including stability indices, were compared with ANOVA followed by Bonferroni post hoc tests. All discrete statistical analyses were performed in SPSS Statistics, version 30, with significance set at *p* < 0.05. Continuous biomechanical data were evaluated in MATLAB version 2023b (Mathworks, Natick, MA, USA) using non-parametric SPM1d repeated-measures ANOVA [39].

SPM1d controls for temporal correlation across the movement cycle. Bonferroni correction (*p* = 0.0085) was applied to account for multiple post hoc comparisons across taping conditions, consistent with recommendations for non-parametric paired testing [39].

Alpha-level thresholding (*p* < 0.05) was applied within the SPM1d framework. This procedure produces a one-dimensional F-test (ANOVA) curve over the duration of the movement cycle (0–100%). Each time point on the curve corresponds to an F-statistic value. When a value exceeds the critical threshold, that region of the curve is considered significantly different from the others.

## 3. Results

### 3.1. Landing Force

**Figure 2:**
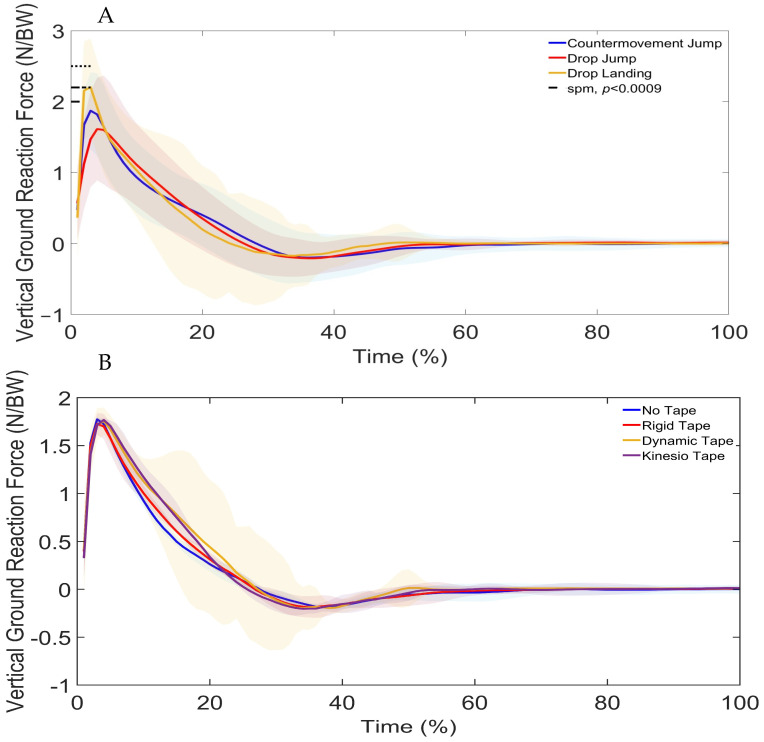
SPM1d Comparison of Landing Force Between (**A**)—Drop Landing (DL), Drop Jump Landing (DJL), and Countermovement Jump (CMJ) and (**B**)—No Tape (NT), Rigid Tape (RT), Dynamic Tape (DT), and Kinesio Tape (KT). Shaded regions indicate the variability around the mean/median. Dotted lines indicate phases where significant differences were detected by SPM1d (*p* < 0.0085).

SPM1d revealed significant differences in ground reaction forces (GRF) between landing types (DL, DJL, and CMJ). Differences were observed at 0–3% (*p* = 0.026) and 15–25% (*p* < 0.001) of the movement cycle (Figure 2A). Post hoc tests showed further differences where the DL showed greater landing forces than the DJL and CMJ, and the CMJ was greater than the DJL (Table 1). Significant differences in GRF were also found between taping conditions (NT, RT, DT, and KT) at 10–12% (*p* = 0.03) of the cycle (Figure 2B). Finally, an interaction between landing type and taping condition was observed at 10% of the movement cycle (*p* = 0.046), indicating that the effect of tape on GRF varied depending on the landing type.

No significant differences were found in peak landing forces across DL, DJL, and CMJ under NT (*p* = 0.24; η^2^ = 0.05, small effect) or RT (*p* = 0.15; η^2^ = 0.07, small effect) (Table 2). However, DT (*p* = 0.02; η^2^ = 0.13, small effect) and KT (*p* = 0.03; W = 0.19, small effect) conditions both showed significantly reduced peak landing forces in DJL compared with DL. These effect sizes indicate that while differences were statistically significant, the practical magnitude of change was modest, suggesting that taping may only provide limited biomechanical benefit in attenuating peak forces.

### 3.2. Stability Indices

Under NT (Table 3), DPSI (*p* < 0.001; η^2^ = 0.631, large effect) and VSI (*p* < 0.001; η^2^ = 0.522, large effect) were significantly greater in DJL and CMJ compared to DL, indicating reduced stability in more complex tasks. APSI (*p* < 0.001; w = 0.657, large effect) was lower in DJL (*p* = 0.002) and CMJ (*p* < 0.001), while MLSI (*p* = 0.005; w = 0.277, small effect) was greater in DJL compared with DL (*p* = 0.004).

In the RT condition, TTS (*p* = 0.02, w = 0.23, small effect) was significantly shorter in DJL compared to DL (*p* = 0.005). DPSI (*p* < 0.001, η^2^ = 0.476, moderate effect) and VSI (*p* < 0.001, η^2^ = 0.507, large effect) remained greater in the DJL and CMJ relative to the DL (*p* < 0.001), while APSI (*p* < 0.001, w = 0.673, large effect) was lower and MLSI (*p* < 0.001, w = 0.767, large effect) greater.

DT showed similar patterns, with reduced TTS (*p* = 0.042, w = 0.176, small effect) in the DJL compared to DL (*p* = 0.037), higher DPSI (*p* < 0.001, η^2^ = 0.311, moderate effect) and VSI (*p* < 0.001, w = 0.332, moderate effect) in DJL and CMJ (*p* ≤ 0,001), and lower APSI (*p* < 0.001, w = 0.756, large effect).

In the KT condition, TTS (*p* = 0.03, w = 0.194, small effect) was shorter in DJL compared to DL (*p* = 0.037). DPSI (*p* < 0.001, w = 0.804, large effect) and VSI (*p* < 0.001, w = 0.806, large effect) were greater in DJL and CMJ compared to DL, while APSI (*p* < 0.001, w = 0.618, large effect) was lower. MLSI (*p* < 0.001, w = 0.576, large effect) was significantly greater in DJL (*p* < 0.001) and CMJ (*p* = 0.004) relative to the DL.

Within-task comparisons showed fewer differences (Table 3). During DL, DT produced higher DPSI (*p* < 0.001; η^2^ = 0.196, small effect) and VSI (*p* < 0.001; η^2^ = 0.192, small effect) than NT, and APSI (*p* < 0.01, η^2^ = 0.266, small effect) differed between DT and NT (*p* < 0.001), DT and RT (*p* = 0.006), and DT and KT (*p* = 0.005). In DJL, only TTS (*p* = 0.012; w = 0.187, small effect) differed, with KT showing lower values than NT (*p* = 0.012). In CMJ, MLSI differed (*p* = 0.005; w = 0.229, small effect), with DT showing greater values than NT (*p* = 0.01), RT (*p* = 0.034), and KT (*p* = 0.023).

### 3.3. Joint Angles

**Figure 3:**
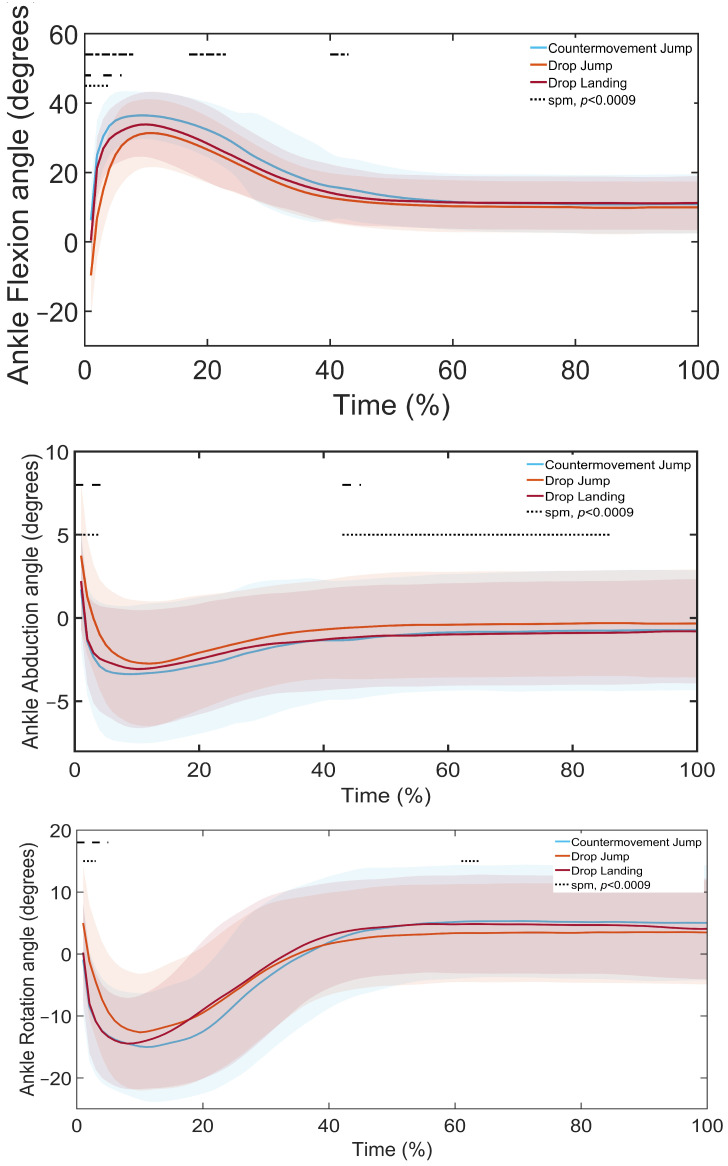
SPM1d Comparison of Ankle Joint Angles during a Drop Landing (DL), Drop Jump Landing (DJL), and Countermovement Jump (CMJ): Shaded regions indicate the variability around the mean/median. Dotted lines indicate phases where significant differences were detected by SPM1d (*p* < 0.0085).

**Figure 4:**
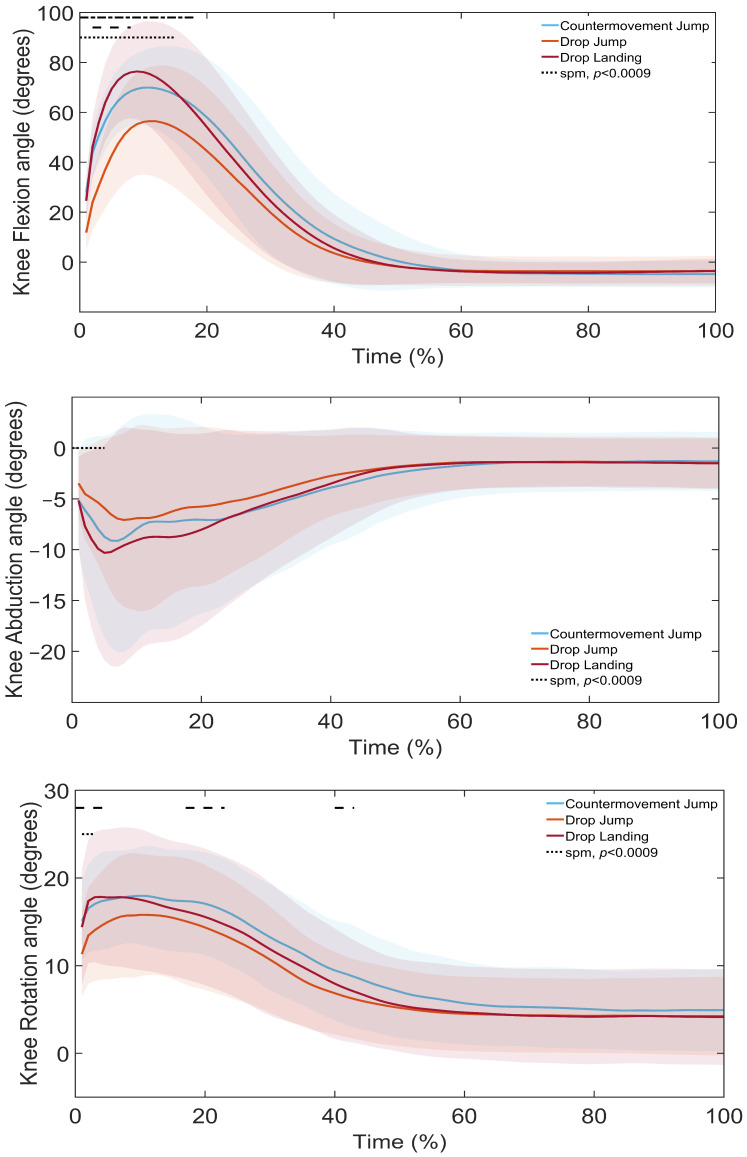
SPM1d Comparison of Knee Joint Angles during a Drop Landing (DL), Drop Jump Landing (DJL), and Countermovement Jump (CMJ): Shaded regions indicate the variability around the mean/median. Dotted lines indicate phases where significant differences were detected by SPM1d (*p* < 0.0085).

**Figure 5:**
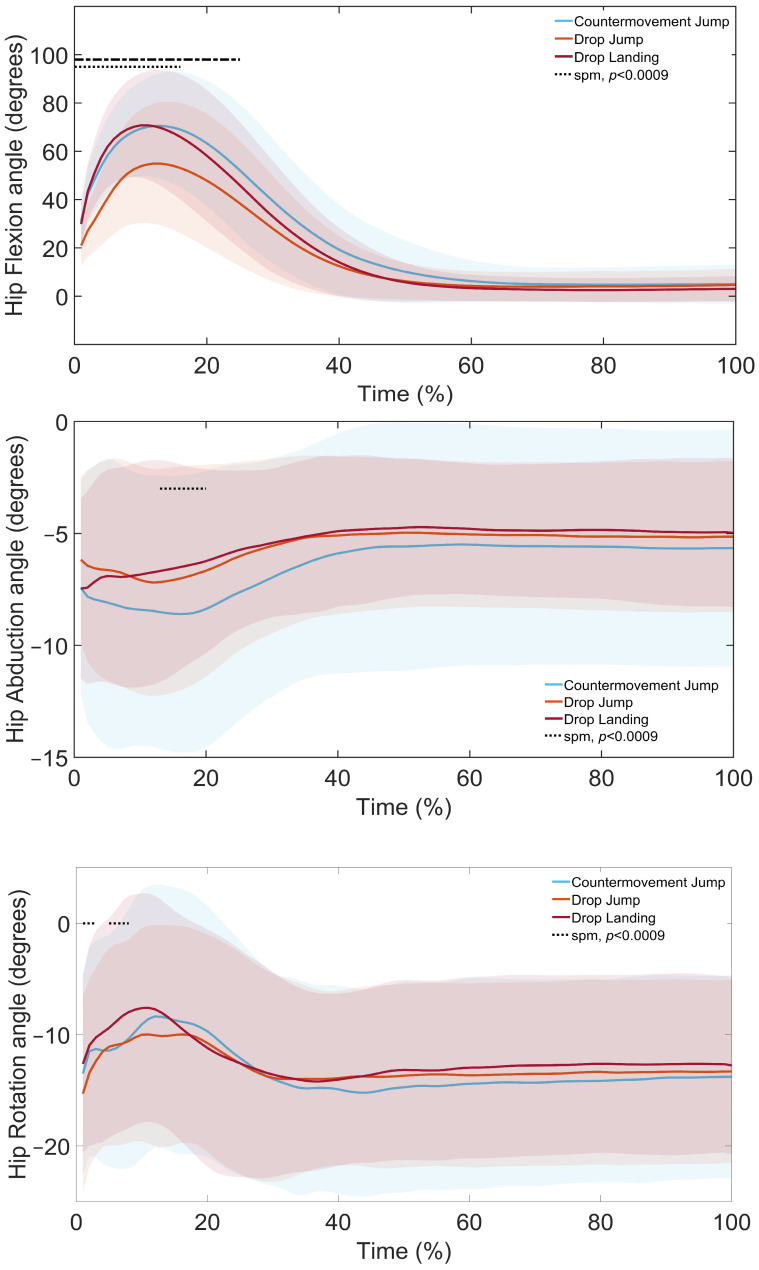
SPM1d Comparison of Hip Joint Angles during a Drop Landing (DL), Drop Jump Landing (DJL), and Countermovement Jump (CMJ): Shaded regions indicate the variability around the mean/median. Dotted lines indicate phases where significant differences were detected by SPM1d (*p* < 0.0085).

SPM1d analysis revealed significant differences in lower limb kinematics across landing types (Figure 3, Figure 4 and Figure 5, Table 4, Table 5 and Table 6). Post hoc comparisons indicated that, at the ankle (Figure 3, Table 4), the DL showed greater flexion, abduction, and rotation than the DJL in the loading and control phases. Similarly, the CMJ demonstrated greater flexion, abduction, and rotation than the DJL throughout the landing phases. Finally, the CMJ had greater flexion than the DL in the loading phase.

At the knee (Figure 4, Table 5), the DL showed greater flexion, abduction, and rotation than the DJL in the loading to attenuation phases, and greater flexion compared to the CMJ in the loading phase. The CMJ also showed greater flexion and rotation than the DJL throughout the landing phases.

At the hip (Figure 5, Table 6), the DL demonstrated greater flexion and rotation than the DJL in the loading to attenuation phases. The CMJ caused greater flexion and abduction than the DL in the attenuation and control phases, as well as greater flexion compared to the DJL in the loading to attenuation phases.

**Figure 6:**
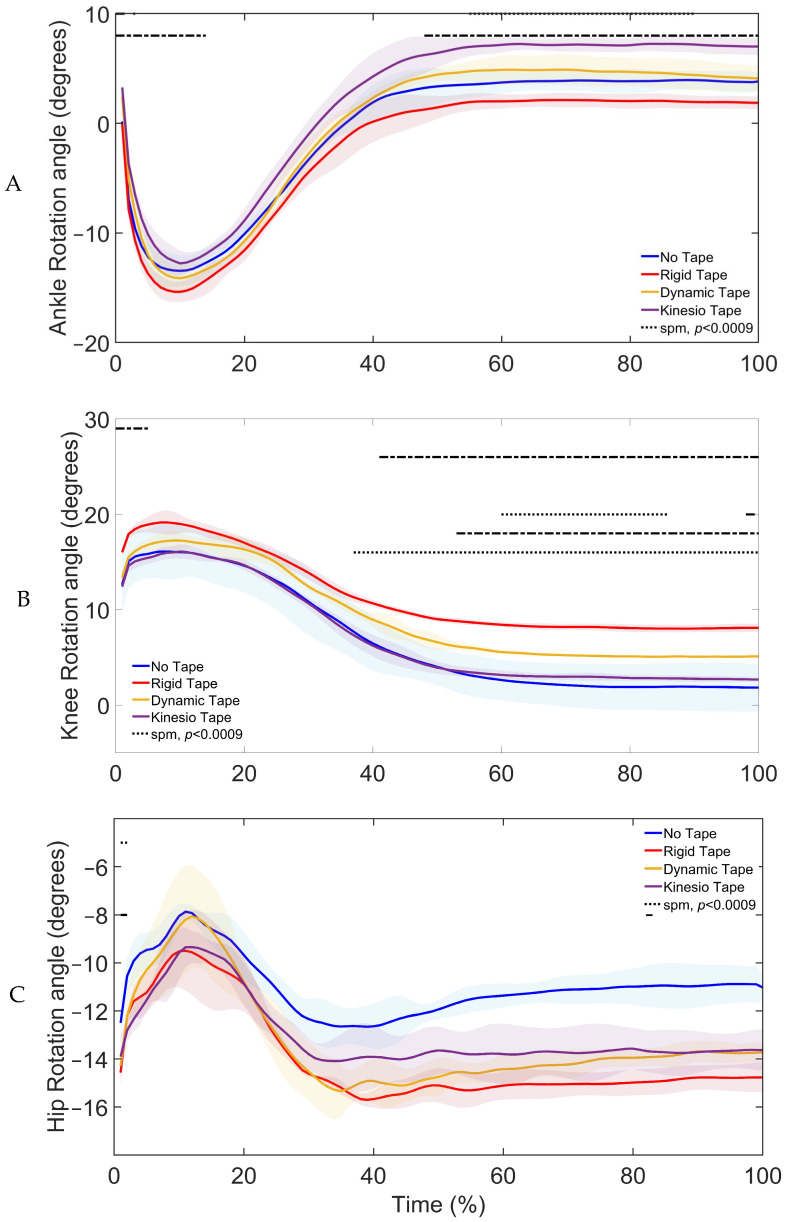
SPM1d Comparisons of (**A**) Ankle Rotation, (**B**) Knee Rotation, and (**C**) Hip Rotation Angle Changes Upon Landing Under the Conditions of No Tape (NT), Rigid Tape (RT), Dynamic Tape (DT), and Kinesio Tape (KT): Shaded regions indicate the variability around the mean/median. Dotted lines indicate phases where significant differences were detected by SPM1d (*p* < 0.0085).

SPM1d analysis of taping conditions (Figure 6) revealed significant differences in ankle (0–8%, 42–100%), knee (0–5%, 28–100%), and hip (76–85%, 95–98%) rotation.

Post hoc testing showed that at the ankle (Figure 6A, Table 7), NT and RT showed more rotation than KT, and RT caused more rotation than DT. At the knee (Figure 6B, Table 7), RT and DT caused more rotation than NT, and RT caused more rotation than DT and KT. At the hip (Figure 6C, Table 7), KT and RT decreased rotation compared to NT.

## 4. Discussion

This study aimed to investigate the effects of different landing types and knee joint taping conditions on lower limb joint stability, kinetics and kinematics. Significant changes were observed in landing forces and stability indices across landing types, taping conditions and their interaction. SPM1d and post hoc analyses of joint kinematics further revealed important changes in ankle, knee and hip joint angles throughout the movement cycles.

The study identified clear differences in GRF, stability indices and lower limb joint kinematics across the DL, DJL and CMJ. Vertical GRF differed significantly between all landing types, with the largest differences observed between the DL and both DJL and CMJ. Stability indices also varied, with overall dynamic stability measured by DPSI and VSI greater in the DJL and CMJ compared to the DL, APSI lower in the DJL and CMJ, and MLSI greater in the DJL compared to the DL. Kinematic comparisons revealed distinct joint strategies. The DL demonstrated greater ankle, knee and hip flexion, as well as increased knee and hip internal rotation than the DJL. The CMJ showed greater ankle and hip flexion than the DL. In the frontal plane, the DL displayed greater knee abduction than both the CMJ and DJL, with the CMJ exceeding the DJL.

The DL, as a single-task landing, requires rapid deceleration of vertical velocity and therefore produces higher GRFs [7,9,10,11]. Higher landing heights are also associated with increased GRFs [1,40], where forces are dissipated through larger joint excursions. In this study, landing heights differed, since the DL height was determined by the 40 cm box, while the DJL and CMJ heights depended on jump performance. The greater ankle, knee and hip flexion, along with increased knee and hip internal rotation in the loading phase of the DL compared with DJL, likely reflects these height-related differences and the associated GRFs [7,9,11,16]. A similar pattern was evident when comparing the CMJ with DJL, suggesting that the higher GRFs in the CMJ are also managed through increased joint motion in the loading phase of the landing, supporting energy dissipation and force attenuation. Although taping conditions significantly reduced peak landing forces in DJL compared with DL, the effect sizes were small (η^2^ = 0.13 for DT; w = 0.19 for KT). This suggests that the practical impact of taping on landing force attenuation was modest. While these small changes may not dramatically alter single landings, they could accumulate across repeated exposures to reduce joint loading risk over time.

Contrary to previous findings [9,16], the DL in this study displayed greater frontal-plane knee abduction than the DJL. One explanation is the absence of a preparatory jump in the DL, which reduces neuromuscular preactivation and increases reliance on passive joint structures for impact absorption, leading to more knee-dominant mechanics [7]. By comparison, the CMJ demonstrated greater knee abduction than the DJL, which is likely due to the increased multiplanar demands of the task [14,15,16].

Dynamic postural stability findings also reflect task complexity. The DL achieved superior overall stability, indicated by lower DPSI and VSI compared with the DJL and CMJ. This likely reflects the reduced neuromuscular demand of a single-task landing [5,6] together with the higher GRFs and the stabilising influence of greater joint flexion, lowering the CoM [9,11]. By contrast, DJL and CMJ incorporated a preparatory jump that engaged neuromuscular preactivation before ground contact [8,12]. This anticipatory activation may enhance forward-backwards control, explaining the lower APSI values in these tasks [1,2]. However, the DJL demonstrated poorer mediolateral stability, as shown by higher MLSI, which is consistent with the combined demands of dropping and rebounding within a single cycle. Taken together, these findings suggest that the DL achieves stability primarily through joint flexion and vertical control, whereas the more complex DJL and CMJ rely on neuromuscular preactivation to attenuate forces across multiple planes, a strategy that may improve forward-backwards control but contributes to greater dynamic instability overall.

Taping further modulated landing mechanics. Both DT and KT lowered landing forces and TTS during the DJL compared to the DL, suggesting that elastic and proprioceptive support enhanced force absorption and facilitated faster postural control under higher task demands [17,18,19]. RT also contributed to faster postural recovery than NT, as reflected in reduced TTS in the DJL, most likely due to the greater joint support it provides [17]. However, reductions in TTS across taping conditions were associated with small effect sizes (w ≈ 0.18–0.23), suggesting limited practical impact on recovery time. Nevertheless, even small improvements in stabilisation speed may benefit athletes during repeated or fatigued landings where neuromuscular control is compromised. Increased internal knee rotation with DT and RT was consistent with previous findings [30,41] and reflects the influence of Mulligan’s taping technique [17,20], which may have enabled the knee to accommodate rotational forces more effectively.

Joint kinematics also varied across taping conditions. Specifically, DT promoted greater ankle and knee rotation [41,42], KT reduced hip rotation relative to NT [19], and RT increased knee rotation [19,20] in the loading and control phases of the landing. These tape-induced adaptations paralleled the task-dependent joint strategies observed across landing types, where increased joint motion supported force dissipation in the loading phase and stability in the control phase of the landings [7,9,10,11].

Overall, these findings indicate that landing strategies are strongly task-dependent. The DL appears optimised for stability and force absorption, the DJL for reactive strength and rapid stabilisation, and the CMJ for explosive power under multiplanar demands. Taping modified these strategies in ways consistent with the mechanical and proprioceptive properties of each tape, supporting its potential role in enhancing postural control and attenuating landing forces in demanding conditions.

The task-dependent landing strategies observed in this study carry important implications for both injury prevention and athletic performance. The DL was associated with greater joint flexion and improved vertical stability, suggesting it may represent a safer landing pattern through enhanced energy dissipation and a lowered CoM. However, the increased knee abduction noted in DL highlights the need to address frontal plane control, as excessive valgus motion is a known risk factor for ACL injury [43]. In contrast, the DJL and CMJ, which are more representative of sport-specific, dual-task movements, were associated with reduced overall stability but enhanced anteroposterior control. These tasks demand rapid transitions between force generation and absorption, increasing neuromuscular load and instability risk. The ability of knee taping to shorten TTS and reduce impact forces during these landings suggests that external support may provide a protective mechanism under high-demand conditions by enhancing proprioception and moderating joint loading [20,42]. Such effects may be particularly valuable in fatigued or high-intensity scenarios, when reactive landings are most common and the risk of injury is greatest.

From a performance perspective, these adaptations highlight the differing priorities of each task. The DL facilitates controlled energy dissipation, while DJL and CMJ promote reactive strength and explosive power.

From a practical perspective, these findings suggest that tape selection may be tailored to task demands. Elastic tapes (KT and DT) appear beneficial in reducing impact forces and facilitating faster postural recovery during reactive landings such as the DJL and CMJ, which dominate match play scenarios. By contrast, RT may be more suitable when stability is prioritised over mobility. Practitioners should therefore consider both the mechanical and situational demands of play when applying taping strategies, integrating them with broader injury prevention and performance programmes.

This study has several limitations. First, the participant sample consisted only of female athletes, which limits generalisability to male players. Second, although taping was applied consistently by a trained practitioner, full blinding was not possible given the distinct appearance of each tape, and participant awareness may have influenced responses. Third, as testing was confined to controlled laboratory conditions, caution should be taken when extrapolating these findings to competitive match play. Although controlled tasks provided clear insights into task and taping specific adaptations, they do not fully replicate the unpredictable and multidirectional landings in match play. Real-world landings often occur under fatigue, opponent contact, and constrained visual fields, which may alter the biomechanical strategies observed here. As such, while these findings provide a mechanistic basis for taping effects, caution should be exercised when generalising to complex game environments. Finally, while the observed adaptations from taping in this study are consistent with proposed mechanical and neurophysiological mechanisms, these interpretations should be considered tentative, as direct measures of muscle activation or proprioceptive feedback were not collected.

## 5. Conclusions

Differences observed between the DL, DJL, and CMJ reflect how increasing task complexity alters the kinetic and kinematic landing strategies. Notably, the taping conditions (RT, DT, and KT) produced more favourable outcomes in reducing landing impact forces and shortening TTS during the more complex tasks (DJL and CMJ) than during the DL. These findings highlight the potential of knee joint taping to enhance force attenuation and postural recovery under increased neuromuscular demands.

Future studies should extend these findings by including male athletes to improve generalisability, employing EMG and proprioception-specific measures to directly examine underlying mechanisms, and evaluating taping effects during real-life sports situations. Such studies would strengthen the understanding of both the protective and performance-related roles of taping in dynamic sporting environments.

## Figures and Tables

**Table 1:** Significant Landing Force Differences Across Landing Tasks Drop Landing (DL), Drop Jump Landing (DJL), and Countermovement Jump (CMJ).

Condition	Landing Comparison	Phase (%)	*p*-Value
NT	DL > DJL	0–3%	0.0004
NT	DL > CMJ	0–2%; 18–25%	0.0009; 0.000002
NT	DJL < CMJ	0–3%	0.0007

**Table 2:** Landing height and forces during a Drop Landing, Drop Jump Landing, and Countermovement Jump between No Tape, Rigid Tape, Dynamic Tape, and Kinesio Tape.

	DL				DJL *				CMJ			
	NT	RT	DT	KT	NT	RT	DT	KT	NT	RT	DT	KT
Peak Landing force (N/BW)	2.28 ±0.62	2.17 ±0.57	2.34 ±0.64	2.30 ±0.68	1.95 ±0.62	1.78 ±0.75	1.79 ±0.67 †	1.92 ±0.68 †	2.04 ±0.60	1.93 ±0.48	2.01 ±0.48	2.02 ±0.58

* Differences across landing conditions: Friedman’s test, *p* < 0.05. † significantly different from DL.

**Table 3:** Comparisons of Time to Stabilisation and Stability Indices Across Landing Types (Drop Landing, Drop Jump Landing, Countermovement Jump) and Taping Conditions (No Tape, Rigid Tape, Dynamic Tape, Kinesio Tape).

	Drop Landing				Drop Jump				Countermovement Jump			
	NT	RT	DT	KT	NT	RT	DT	KT	NT	RT	DT	KT
TTS *	1.49 ±0.28	1.54 ±0.33	1.44 ±0.28	1.43 ±0.34	1.51 ±0.36	1.33 ±0.34 †	1.27 ±0.36 †	1.19 ±0.30 †	1.45 ±0.25	1.51 ±0.29	1.43 ±0.23	1.40 ±0.23
DPSI *	0.26 ±0.06	0.30 ±0.05	0.37 ±0.11	0.31 ±0.07	0.59 ±0.18 †	0.57 ±0.15 †	0.58 ±0.17 †	0.62 ±0.19 †	0.51 ±0.14 †	0.52 ±0.14 †	0.54 ±0.15 †	0.56 ±0.16 †
VSI *	0.25 ±0.06	0.29 ±0.06	0.36 ±0.10	0.30 ±0.07	0.59 ±0.18†	0.58 ±0.15 †	0.58 ±0.17 †	0.62 ±0.19 †	0.51 ±0.14 †	0.52 ±0.14 †	0.54 ±0.15 †	0.56 ±0.16 †
APSI *	0.06 ±0.01	0.06 ±0.01	0.08 ±0.01	0.06 ±0.01	0.03 ±0.03 †	0.03 ±0.03 †	0.02 ±0.02 †	0.04 ±0.05 †	0.02 ±0.01 †	0.02 ±0.01 †	0.02 ±0.004 †	0.02 ±0.01 †
MLSI *	0.01 ±0.01	0.02 ±0.01	0.03 ±0.02	0.02 ±0.01	0.03 ±0.01 †	0.03 ±0.01 †	0.05 ±0.07	0.06 ±0.07 †	0.03 ±0.02	0.03 ±0.01 †	0.04 ±0.01	0.03 ±0.01 †

* Differences across landing conditions: Friedman’s test, *p* < 0.05. † significantly different from DL.

**Table 4:** Significant Ankle Joint Kinematic Differences Across Landing Tasks (Drop Landing, Drop Jump Landing, and Countermovement Jump).

Joint	Joint Action	Landing Comparison	Phase (%)	*p*-Value
Ankle	Flexion	DL > DJL	0–4%	*p* = 0.00069
		DL < CMJ	0–1%; 3–6%	*p* = 0.00089; *p* = 0.00068
		DJL < CMJ	0–8%; 17–23%; 40–43%	*p* = 0.00016; *p* = 0.00042; *p* = 0.00082
	Abduction	DL > DJL	0–4%; 43–89%	*p* = 0.00073; *p* < 0.000001
		DJL < CMJ	0–5%; 32–46%	*p* = 0.00057; *p* = 0.00028
	Rotation	DL > DJL	0–5%	*p* = 0.00052
		DL < DJL	61–64%	*p* = 0.00083
		DJL < CMJ	0–5%	*p* = 0.00057

**Table 5:** Significant Knee Joint Kinematic Differences Across Landing Tasks (Drop Landing, Drop Jump Landing, and Countermovement Jump).

Joint	Joint Action	Landing Comparison	Phase (%)	*p*-Value
Knee	Flexion	DL > DJL	0–15%	*p* = 0.00002
		DL > CMJ	2–8%	*p* = 0.00049
		DJL < CMJ	0–18%	*p* = 0.000004
	Abduction	DL > DJL	0–5%	*p* = 0.00041
	Rotation	DL > DJL	1–3%	*p* = 0.00086
		DJL < CMJ	0–5%; 17–23%; 32–46%	*p* = 0.00055; *p* = 0.00042; *p* = 0.000004

**Table 6:** Significant Hip Joint Kinematic Differences Across Landing Tasks (Drop Landing, Drop Jump Landing, and Countermovement Jump).

Joint	Joint Action	Landing Comparison	Phase (%)	*p*-Value
Hip	Flexion	DL > DJL	0–16%	*p* = 0.00040
		DL < CMJ	73–77%	*p* = 0.000693
		DJL < CMJ	0–25%	*p* = 0.000000198
	Abduction	DL < CMJ	13–20%	*p* = 0.000505
	Rotation	DL > DJL	1–3%; 5–8%	*p* = 0.000709; *p* = 0.00071

**Table 7:** Significant Lower Limb Joint Rotation Differences Across Taping Conditions (No Tape, Rigid Tape, Dynamic Tape, Kinesio Tape).

Joint	Joint Action	Landing Comparison	Phase (%)	*p*-Value
Ankle	Rotation	NT > KT	55–90%	*p* < 0.000001
		RT > DT	0–3%	*p* = 0.0008
		RT > KT	0–14%; 48–100%	*p* = 0.0001; *p* < 0.000001
Knee	Rotation	NT < DT	60–86%; 98–100%	*p* = 0.0000012; *p* = 0.0009
		NT < RT	41–100%	*p* < 0.000001
		RT > DT	53–100%	*p* < 0.000001
		RT > KT	33%; 37–100%	*p* = 0.0009; *p* < 0.000001
Hip	Rotation	NT > KT	0–5%	*p* = 0.00087
		NT > RT	1–2%; 82–83%	*p* = 0.0009; *p* = 0.0009

## Data Availability

The original contributions presented in this study are included in the article. Further inquiries can be directed to the corresponding author.

## Abbreviations

The following abbreviations are used in this manuscript:

APSI	Anteroposterior stability index
CMJ	Countermovement jump
CoM	Centre of mass
DL	Drop landing
DJL	Drop jump landing
DPS	Dynamic postural stability
DPSI	Dynamic postural stability index
DT	Dynamic tape
GRF	Ground reaction force
KT	Kinesio tape
MLSI	Mediolateral stability index
NT	No tape
RT	Rigid tape
RSI	Reactive strength index
SPM1d	One-dimensional statistical parametric mapping
TTS	Time to stabilisation
VSI	Vertical stability index

## References

[1] Toussaint T.D., Schepens B. (2024). Biomechanical behavior of the lower limbs and of the joints when landing from different heights. J. Biomech..

[2] Gambelli C.N., Theisen D., Willems P.A., Schepens B. (2015). Motor Control of Landing from a Jump in Simulated Hypergravity. PLoS ONE.

[3] Niu W., Zhang M., Fan Y., Zhao Q. (2013). Dynamic postural stability for double-leg drop landing. J. Sports Sci..

[4] Hovey S., Wang H., Judge L.W., Avedesian J.M., Dickin D.C. (2021). The effect of landing type on kinematics and kinetics during single-leg landings. Sports Biomech..

[5] Giesche F., Wilke J., Engeroff T., Niederer D., Hohmann H., Vogt L., Banzer W. (2020). Are biomechanical stability deficits during unplanned single-leg landings related to specific markers of cognitive function?. J. Sci. Med. Sport.

[6] Lin J.Z., Tai W.H., Chiu L.Y., Lin Y.A., Lee H.J. (2020). The Effect of Divided Attention with Bounce Drop Jump on Dynamic Postural Stability. Int. J. Sports Med..

[7] Chang J.S., Nam S.M. (2024). Biomechanical Comparison of Lower Extremities between Drop Landing and Landing after Jumping. PNF Mov..

[8] Ishida T., Koshino Y., Yamanaka M., Ueno R., Taniguchi S., Samukawa M., Saito H., Matsumoto H., Aoki Y., Tohyama H. (2018). The effects of a subsequent jump on the knee abduction angle during the early landing phase. BMC Musculoskelet. Disord..

[9] Mache M.A., Hoffman M.A., Hannigan K., Golden G.M., Pavol M.J. (2013). Effects of decision making on landing mechanics as a function of task and sex. Clin. Biomech..

[10] Chiu L.Z.F., Moolyk A.N. (2015). Segment Kinematics Differ Between Jump and Drop Landings Regardless of Practice. J. Appl. Biomech..

[11] Harry J.R., Freedman Silvernail J., Mercer J.A., Dufek J.S. (2018). Bilateral Comparison of Vertical Jump Landings and Step-off Landings from Equal Heights. J. Strength Cond. Res..

[12] Afifi M., Hinrichs R.N. (2012). A Mechanics Comparison Between Landing from a Countermovement Jump and Landing from Stepping Off a Box. J. Appl. Biomech..

[13] Harry J.R., Lanier R., Nunley B., Blinch J. (2019). Focus of attention effects on lower extremity biomechanics during vertical jump landings. Hum. Mov. Sci..

[14] Almonroeder T.G., Kernozek T., Cobb S., Slavens B., Wang J., Huddleston W. (2018). Cognitive Demands Influence Lower Extremity Mechanics During a Drop Vertical Jump Task in Female Athletes. J. Orthop. Sports Phys. Ther..

[15] Monfort S.M., Pradarelli J.J., Grooms D.R., Hutchison K.A., Onate J.A., Chaudhari A.M.W. (2019). Visual-Spatial Memory Deficits Are Related to Increased Knee Valgus Angle During a Sport-Specific Sidestep Cut. Am. J. Sports Med..

[16] Letchford E.C. (2020). The Second Landing from a Drop Vertical Jump: A Biomechanical Analysis and Clinical Application to Enhance Evaluation of ACL Injury Risk in Female Athletes. Ph.D. Thesis.

[17] McNeill W., Pedersen C. (2016). Dynamic tape. Is it all about controlling load?. J. Bodyw. Mov. Ther..

[18] Silva R.O., Carlos F.R., Morales M.C., Emerick V.d.S., Teruyu A.I., Valadão V.M.A., Carvalho L.C., Lobato D.F. (2021). Effect of two Dynamic Tape^TM^ applications on the electromyographic activity of the gluteus medius and functional performance in women: A randomized, controlled, clinical trial. J. Bodyw. Mov. Ther..

[19] Limroongreungrat W., Boonkerd C. (2019). Immediate effect of ACL kinesio taping technique on knee joint biomechanics during a drop vertical jump: A randomized crossover controlled trial. BMC Sports Sci. Med. Rehabil..

[20] Rosen A.B., Ko J., Brown C.N. (2017). Single-limb landing biomechanics are altered and patellar tendinopathy related pain is reduced with acute infrapatellar strap application. Knee.

[21] Rajasekar S., Kumar A., Patel J., Ramprasad M., Samuel A.J. (2018). Does Kinesio taping correct exaggerated dynamic knee valgus? A randomized double blinded sham-controlled trial. J. Bodyw. Mov. Ther..

[22] Saki F., Romiani H., Ziya M., Gheidi N. (2022). The effects of gluteus medius and tibialis anterior kinesio taping on postural control, knee kinematics, and knee proprioception in female athletes with dynamic knee valgus. Phys. Ther. Sport.

[23] Yona T., Kamel N., Cohen-Eick G., Ovadia I., Fischer A. (2024). One-dimension statistical parametric mapping in lower limb biomechanical analysis: A systematic scoping review. Gait Posture.

[24] Honert E.C., Pataky T.C. (2021). Timing of gait events affects whole trajectory analyses: A statistical parametric mapping sensitivity analysis of lower limb biomechanics. J. Biomech..

[25] Slovák L., Zahradník D., Land W.M., Sarvestan J., Hamill J., Abdollahipour R. (2024). Response of Knee Joint Biomechanics to Landing Under Internal and External Focus of Attention in Female Volleyball Players. Mot. Control.

[26] Zhang Z., Xu D., Gao X., Zhou H., Baker J.S., Radak Z., Gu Y. (2025). Differences of simulated ankle dorsiflexion limitation on lower extremity biomechanics during long jump takeoff. Heliyon.

[27] Bi G., Hua L., Sun J., Xu Q., Li G. (2024). Impact of different landing heights on the contact force in the medial tibiofemoral compartment and the surrounding muscle force characteristics in drop jumps. PLoS ONE.

[28] Hughes S., Warmenhoven J., Haff G.G., Chapman D.W., Nimphius S. (2022). Countermovement Jump and Squat Jump Force-Time Curve Analysis in Control and Fatigue Conditions. J. Strength Cond. Res..

[29] De Ridder R., Willems T., Vanrenterghem J., Roosen P. (2015). Effect of Tape on Dynamic Postural Stability in Subjects with Chronic Ankle Instability. Int. J. Sports Med..

[30] Szeles K.T., Green A. (2025). Strapping for knee stability: Kinetic and kinematic comparisons of dynamic, rigid, and kinesio taping. J. Phys. Educ. Sport.

[31] Hendry D., Campbell A., Ng L., Grisbrook T.L., Hopper D.M. (2015). Effect of Mulligan’s and Kinesio knee taping on adolescent ballet dancers knee and hip biomechanics during landing. Scand. J. Med. Sci. Sports.

[32] Howe A., Campbell A., Ng L., Hall T., Hopper D. (2015). Effects of two different knee tape procedures on lower-limb kinematics and kinetics in recreational runners. Scand. J. Med. Sci. Sports.

[33] Torres R., Trindade R., Gonçalves R.S. (2016). The effect of kinesiology tape on knee proprioception in healthy subjects. J. Bodyw. Mov. Ther..

[34] Whyte E.F., Kennelly P., Milton O., Richter C., O’Connor S., Moran K.A. (2017). The effects of limb dominance and a short term, high intensity exercise protocol on both landings of the vertical drop jump: Implications for the vertical drop jump as a screening tool. Sports Biomech..

[35] Thomas C., Ismail K.T., Simpson R., Comfort P., Jones P.A., Dos’Santos T. (2019). Physical Profiles of Female Academy Netball Players by Position. J. Strength Cond. Res..

[36] Harry J.R., Simms A., Hite M. (2024). Establishing Phase Definitions for Jump and Drop Landings and an Exploratory Assessment of Performance-Related Metrics to Monitor During Testing. J. Strength Cond. Res..

[37] Ross S.E., Guskiewicz K.M. (2003). Time to Stabilization: A Method for Analyzing Dynamic Postural Stability. Athl. Ther. Today.

[38] Wikstrom E.A., Tillman M.D., Smith A.N., Borsa P.A. (2005). A new force-plate technology measure of dynamic postural stability: The dynamic postural stability index. J. Athl. Train..

[39] Pataky T. (2022). Introduction SPM1d 0.4 Documentation. https://spm1d.org/.

[40] Di Giminiani R., Giovannelli A., Capuano L., Izzicupo P., Di Blasio A., Masedu F. (2020). Neuromuscular Strategies in Stretch–Shortening Exercises with Increasing Drop Heights: The Role of Muscle Coactivation in Leg Stiffness and Power Propulsion. Int. J. Environ. Res. Public Health.

[41] Mackay G.J.K., Stearne S.M., Wild C.Y., Nugent E.P., Murdock A.P., Mastaglia B., Hall T.M. (2020). Mulligan Knee Taping Using Both Elastic and Rigid Tape Reduces Pain and Alters Lower Limb Biomechanics in Female Patients with Patellofemoral Pain. Orthop. J. Sports Med..

[42] Wu C.K., Lin Y.C., Chen Y.L., Chao Y.P., Hsieh T.H. (2024). The Influence of Dynamic Taping on Landing Biomechanics after Fatigue in Young Football Athletes: A Randomized, Sham-Controlled Crossover Trial. Bioengineering.

[43] Belcher S., Whatman C., Brughelli M. (2024). A systematic video analysis of 21 anterior cruciate ligament injuries in elite netball players during games. Sports Biomech..

